# 4D-Printed Tool for Compressing a Shape Memory Polyurethane Foam during Programming

**DOI:** 10.3390/polym16101393

**Published:** 2024-05-14

**Authors:** Dilip Chalissery, Thorsten Pretsch

**Affiliations:** Fraunhofer Institute for Applied Polymer Research IAP, Geiselbergstraße 69, 14476 Potsdam, Germany; dilip.chalissery@iap.fraunhofer.de

**Keywords:** 4D printing, shape memory polymer, programmable material, polyurethane, shape memory polymer foam, programming tool

## Abstract

Although several force application concepts are known that can be used to deform shape memory polymers (SMPs) within the scope of programming, controlled deformation is challenging in the case of samples with a cylinder-like shape, which need to be homogeneously compressed starting from the lateral surface. To solve this problem, this contribution follows a material approach that takes advantage of four-dimensional (4D) printing. Fused filament fabrication (FFF) was used as an additive manufacturing (AM) technique to produce a thermoresponsive tool in a cylindrical shape from a polyether urethane (PEU) having a glass transition temperature (*T*_g_) close to 55 °C, as determined by differential scanning calorimetry (DSC). Once it was 4D-printed, a sample of laser cut polyester urethane urea (PEUU) foam with a cylindrical wall was placed inside of it. Subsequent heating to 75 °C and keeping that temperature constant for 15 min resulted in the compression of the foam, because the internal stresses of the PEU were transferred to the PEUU, whose soft segments were completely molten at 65 °C as verified by DSC. Upon cooling to −15 °C and thus below the offset temperature of the soft segment crystallization transition of the PEUU, the foam was fixed in its new shape. After 900 days of storage at temperatures close to 23 °C, the foam recovered its original shape upon reheating to 75 °C. In another experiment, a 4D-printed cylinder was put into hibernation for 900 days before its thermoresponsiveness was investigated. In the future, 4D-printed tools may be produced in many geometries, which fit well to the shapes of the SMPs to be programmed. Beyond programming SMP foams, transferring the forces released by 4D-printed tools to other programmable materials can further expand technical possibilities.

## 1. Introduction

Shape memory polymers (SMPs) belong to the class of smart materials. SMPs can fix a temporary shape after thermomechanical treatment known as “programming” [1,2,3,4,5,6]. Triggered by an external stimulus such as a temperature increase, an SMP returns to its permanent shape. This entropically driven process is defined as a “one-way” shape memory effect. In the last three decades, various programming methods have been developed that commonly involve the deformation of a phase segregated SMP in its elastic state, followed by crystallizing or vitrifying the switching segment and unloading [7,8,9,10,11,12,13,14,15]. However, these procedures often require the use of complex systems with only a limited degree of freedom for the application of force, such as a tensile testing machine with a temperature chamber and the corresponding technical equipment for tensile, compression and three-point bending tests.

Four-dimensional (4D) printing is an emerging additive manufacturing (AM) technique [16,17,18,19,20,21,22,23,24,25,26,27,28,29,30]. The original 4D printing approach as introduced by Skylar Tibbits in 2014 was inspired by a self-assembly concept, emphasizing that printed structures are no longer static but capable of responding to environmental changes by changing their shape [31]. This could be achieved by a folding mechanism, in which a hydrophilic polymer as a water-responsive active component exerted pressure on a rigid counterpart during swelling and thus caused the deformation of the overall structure. The concept was later transferred to the AM technology fused filament fabrication (FFF) by printing thermoresponsive objects [32,33,34,35,36,37,38,39,40,41]. At the molecular level, parameters like nozzle temperature, printing pressure and printing speed influence the orientation of polymer chains. The prerequisite for distinct shape changes is that an extruded polymer strand is placed on a printer’s platform or on a previously deposited layer and quickly cooled to achieve the vitrification of polymer chains. Thereupon internal stresses can be stored in the polymer whose chains are characterized by states of higher order compared with objects obtained from conventional melt-based processing methods, in which the relaxation processes occurred. In the past, 4D printing of higher objects using FFF has sometimes caused drastic relaxation effects, which is probably due to the lack of control over temperature as the number of layers increases, unless additional temperature control devices are used. From a conceptual point of view, selecting a thermoresponsive SMP with a glass transition temperature (*T*_g_) above ambient temperature for 4D printing can ensure that the object remains stable under room temperature conditions until the temperature rises above the switching temperature of the SMP. As soon as the polymer, i.e., a specific type of polyether urethane (PEU), is heated above its *T*_g_, it releases internal stresses through molecular movement driven by a gain in entropy, which leads to macroscopic shrinkage according to the direction of strand deposition. One of our recent studies has demonstrated that the degree of shrinkage of a PEU depends on the processing (nozzle) temperature. In the global pandemic of the coronavirus disease, heat-shrinkable door openers were manufactured, which can undergo mechanical recycling after use; the reusability of the printing material in a later 4D printing with only minor losses in terms of the magnitude of shape change was demonstrated [41]. The same study also showed that regardless of the selected geometry, such as a hollow cylinder, hollow cuboid or solid cuboid, the shrinkage or bending behavior of the printed object was consistent with the direction of deposition of the polymer strands, which were contracting upon heating the print material above its *T*_g_. All this shows that it is possible to manufacture objects with greater height. exceeding e.g., 30 mm, without significantly releasing internal stresses. Another advantage of FFF is that when selecting a 100 µm nozzle, even filigree structures can be manufactured as demonstrated five years ago when printing machine-readable quick response codes using a pristine and a dyed SMP [42].

Today, various force application concepts are known that can be used to deform SMPs within the scope of programming. In addition to manual deformation, these include the use of appropriate machines such as those frequently encountered in the characterization of shape memory properties. Examples comprise a tensile testing machine coupled with a temperature chamber and a dynamic mechanical analysis (DMA) device. While there is virtually no control over the deformation rate in the manual case, this parameter can be easily adjusted, especially with the technical devices. Regardless of the testing machine selected and the corresponding test setup, the application of external forces can lead to stretching, compression, bending or torsion of a material. For instance, during stretching or compression, the force acts vertically on a sample. In this case, it is almost not possible to build up forces that act on the lateral surface of a cylindrical object. To tackle this challenge, 4D printing via FFF was applied to produce a heat-shrinkable tool made from a self-synthesized PEU. In its production, the same material was selected for which a pronounced shrinkage behavior had already been demonstrated after AM [41]. As an object with shape memory properties to be programmed, a self-synthesized, laser cut polyester urethane urea (PEUU) foam was chosen [43]. In order to enable a later fit, the diameter of the largely cylindrical foam sample was selected to be only slightly smaller than the inner diameter of the cylindrical shrink tool. This way, the technological proof of feasibility of programming the SMP foam with the 4D-printed tool by selecting an adequate sequence of heating and cooling steps will be demonstrated. As no data on the long-term durability of 4D-printed objects have been available to our knowledge, this aspect will also be covered in the present work.

## 2. Materials and Methods

*Material*: Polypropylene glycol (PPG)-based PEU was selected as the 4D printing material. According to the prepolymer method, Desmophen^®^ 1262 BD from Covestro Deutschland AG (Leverkusen, Germany), a linear PPG with a molecular weight of about 430 g mol^−1^, was reacted in the first step with 4,4′-diphenylmethane diisocyanate (MDI) from Fisher Scientific (Schwerte, Germany) to build up an isocyanate-endcapped prepolymer. Adjacently, the chain extender 1,4-butanediol (BD) from Alfa Aesar (Kandel, Germany) was added, resulting in the formation of the PEU. The weight ratio of hard to soft segments was about 60:40, corresponding to a molar ratio of the reactants PPG, MDI and BD of 1:2.17:1.16 [41]. In detail, PPG was heated to 125 °C under stirring in the presence of nitrogen, before two droplets of PPG and 5 wt% of titanium (IV) bis(acetylacetonate) diisopropoxide from Merck (Darmstadt, Germany) were added. After the further addition of MDI, the mixture was heated to 155 °C, at which it was stirred for 5 min. While increasing the stirring speed, the obtained prepolymer was brought to react with the chain extender BD. Once the viscosity increased significantly, the melt was poured on a plate that was covered with a thin film of polytetrafluoroethylene. Afterwards, the PEU was placed for 120 min in an oven at a temperature of 80 °C. After curing, the PEU was cryo-milled using a cryogenic mill from Hosokawa Alpine AG (Augsburg, Germany) and processed with a filament, which was then fed into the FFF process.

The second SMP used in this work was a PEUU foam with soft segments built from poly(1,10-decamethylene adipate) diol (PDA) and poly(1,4-butylene adipate) diol (PBA). The foam was obtained via reactive foaming in a one-pot two step reaction using a mixture of PDA and PBA with a ratio of 6.5:1 and a slight excess of MDI with a ratio of NCO/OH = 1.03. For this purpose, the polyester polyols were first placed in a 550 mL polypropylene beaker and dried in a vacuum oven overnight at 85 °C. The polymer melt was removed after 12 h and kept for 2 min while stirring on a heating plate at 80 °C. Adjacently, 1.0 parts per hundred polyol (pphp) of the surfactant Tegostab^®^ B8407 from Evonik (Essen, Germany), 0.3 pphp of the tin-free gelling catalyst K-Kat XC-B221 from Worlée (Hamburg, Germany) and 36.7 pphp of molten MDI were added under vigorous stirring. To finalize the reaction and start the foaming process, 1.0 pphp of BD, 2.0 pphp of diethanolamine from Merck Millipore (Darmstadt, Germany) and a mixture containing 1.3 pphp of deionized water and 0.1 pphp of the blowing catalyst 1,4-diazabicyclo [2.2.2]octane from Alfa Aesar (Kandel, Germany) were added at high stirring speed. Once significant foaming occurred, the stirrer was removed in order to enable the free rise of the foam. To achieve post-curing, the freshly produced foam was stored at 23 °C overnight [43].

*Virtual design and fused filament fabrication*: The AutoCAD 2011 software from Autodesk, Inc. (San Rafael, CA, USA) was used to design an object in the form of a cylinder. The cylinder was characterized in its thermoresponsive state by an inner diameter of 50 mm, an outer diameter of 52 mm and a height of 20 mm. The developed computer-aided design (CAD) model was exported in standard triangle language format and subsequently employed for slicing. Once the design of the cylinder was finalized, Ultimaker Cura version 3.6.1 (2023) [44] served as the slicing software to generate numerically controlled codes, which included machine control instructions like G-codes and M-codes. The 3D model was imported into the program, and the model was sliced into layers according to the predefined printing parameters. The technical setup and main parameters used for 4D printing included a nozzle with an opening of 400 µm which was used for the print head, a nozzle temperature of 168 °C, a printing speed of the print head of 30 mm × s^−1^, a build platform temperature of 23 °C and a layer height of 0.15 mm. To start FFF and thus 4D printing, the generated G-code data file was transferred to the commercially available printer Ultimaker 3.

*Characterization of thermal and thermomechanical properties*: The phase transitions of both PEU and PEUU were characterized using a Q100 DSC device from TA Instruments (New Castle, DE, USA). Dried pieces of filament of PEU weighing approximately 5 mg and a sample of PEUU with a weight of about 3 mg were subjected to the experiments. Initially, the samples were heated from 23 °C to 100 °C before they were cooled to −80 °C. Subsequently, heating from −80 °C to 100 °C was conducted to complete the measurement. For heating and cooling, rates of 10 °C min^−1^ were selected. The samples were held at the minimum and maximum temperatures for 2 min each. The *T*_g_ of the PEU was determined from the second heating as the temperature corresponding to half the step height between the tangents of the baseline, utilizing the standard analysis software of the calorimeter.

The thermomechanical behavior of freshly synthesized PEU, a sample of it after 900 days of storage under room temperature conditions, and a sample of PEUU were investigated with a Q800 DMA from TA Instruments (New Castle, DE, USA). One cuboidal sample of PEU, characterized by the dimensions of 35 mm × 6 mm × 3 mm, was obtained from FFF and clamped in the specimen holder so that the distance between the upper and lower clamp was 17 mm. Specifically, single cantilever clamps operating in multifrequency strain mode were employed.

After reactive foaming, the PEUU foam was cut into cubes measuring 10 mm × 10 mm × 10 mm and one of these samples was fixed in the compression clamps of the DMA device. In advance of each measurement, the sample was cooled to −80 °C and held at this temperature for 5 min. Adjacently, heating to 100 °C was conducted at a rate of 3 °C × min^−1^ followed by a final temperature holding step of 5 min. The most important measurement parameters included a frequency of 10 Hz, a static force of 0.1 N and an oscillating amplitude of 10 µm.

**Figure 1:**
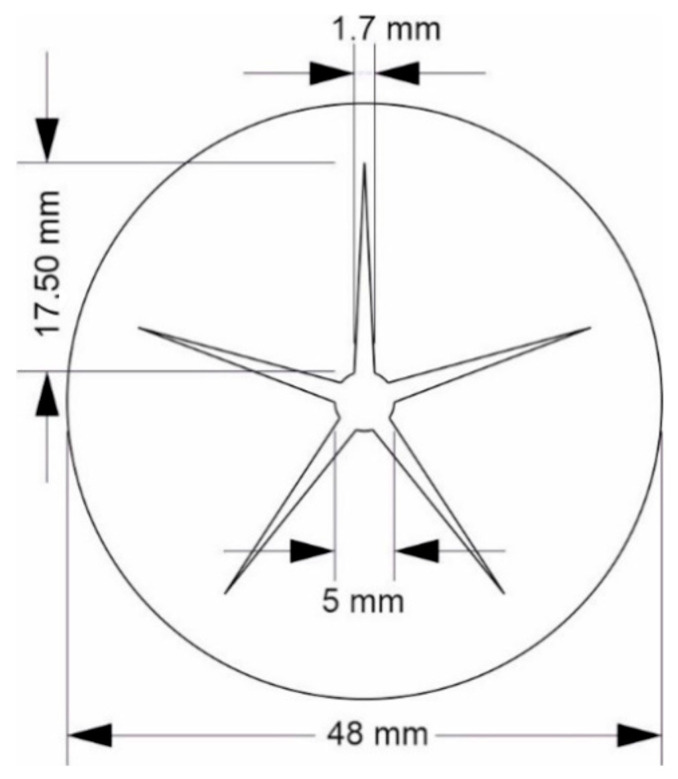
Top view of the design of a sample made of SMP foam, characterized by a circular outer edge and star-shaped cut-out.

*Laser cutting of PEUU foam*: To better illustrate the material behavior in the course of programming and during shape recovery, a cylinder-like foam sample was designed with a star-shaped recess on the inside. The star shape and the outer boundary of the foam, as shown in Figure 1, were created with CorelDraw 2019 (Corel Corp., Ottawa, ON, Canada) [45]. The outer diameter of the foam was set at 48 mm and the height of the foam at 15 mm. A 30 W laser cutter (Epilog Laser, Golden, CO, USA) was used to precisely cut the star and border shapes from a square piece of PEUU foam, measuring 60 mm × 60 mm × 15 mm.

*Programming of PEUU foam*: To follow the deformation behavior of a laser cut sample of PEUU foam (Figure 1), it was encased in the 4D-printed cylinder and heated in a temperature chamber to 75 °C. In regular time intervals, photographs were taken. After 15 min at 75 °C, it was cooled to −15 °C and held there for 15 min before being heated to 23 °C. Adjacently, the outer diameter of the strongly deformed PEU cylinder and its height were determined with a digital calliper.

*Investigation of shape memory properties and long-term stability*: A 4D-printed cylinder made from PEU and a laser cut sample of PEUU foam (Figure 1) were joined together by heating to 75 °C for 15 min, before cooling to −15 °C and heating to 23 °C were applied. The samples were then stored behind a double-glazed insulated window for 900 days. At the end of the storage period, both the 4D-printed cylinder and the foam were separated. Each part was then heated to 75 °C in a thermochamber from MTS Systems Corporation (Eden Prairie, MN, USA) using a rate of 5 °C × min^−1^. Adjacently, the temperature was maintained for 15 min. To evaluate the shape memory properties, the dimensions of the 4D-printed cylinder and the foam were determined with a digital calliper. In another experiment, a 4D-printed cylinder was manufactured and stored for 900 days at temperatures below 30 °C, before its thermoresponsiveness was investigated by heating to 75 °C.

## 3. Results and Discussion

**Figure 2:**
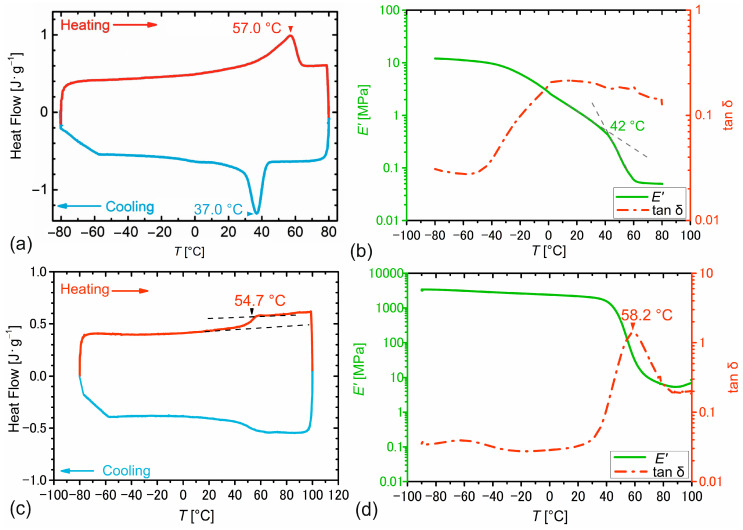
Thermal and thermomechanical properties of PEUU foam (**a**,**b**) and freshly synthesized PEU later used as a 4D printing material (**c**,**d**) as determined by DSC ((**a**,**c**), second heating in red color and cooling in blue color with temperature rates of 10 °C × min^−1^) and DMA ((**b**,**d**), temperature dependence of storage modulus E’, green solid line, and d, loss factor tan δ, red dashed dotted line at a heating rate of 3 °C × min^−1^).

To demonstrate the feasibility of programming PEUU foam with a 4D-printed tool made from PEU, the thermal and thermomechanical properties of both a self-synthesized PEUU and a self-synthesized PEU were examined (Figure 2). The PEUU foam was obtained from reactive foaming [43]. It was characterized by a melting transition determined by DSC in between 20 °C and 65 °C with a melting peak at about 57 °C and a crystallization transition, extending from 45 °C to 0 °C with a crystallization peak at 37 °C (Figure 2a). Both transitions can be assigned to the soft segments of PDA and PBA of the PEUU [43]. In a DMA experiment (Figure 2b), the melting of the soft segments was also indicated by the evolution of the storage modulus. Here, two lines, one associated with a glass transition at lower temperatures and one associated with a melting transition at higher temperatures intersected at 42 °C. The decrease in storage modulus E’ stopped at around 60 °C. It was therefore clear that for the programming of the foam, the temperature should be increased to more than 65 °C to devitrify and melt the soft segments and deform the foam more easily, and that a temperature below 0 °C will be suitable for unloading the solidified foam after cooling due to the crystallization of the soft segments and their partial vitrification.

The material used for 4D printing was a self-synthesized PEU, which had already proven that it was capable of drastically changing its shape after additive manufacturing. Indeed, when heated to at least 65 °C, solid cuboids of the material obtained from FFF were characterized by heat-induced shrinkage of up to 63% after 4D printing [41]. In a DSC measurement, the phase segregated PEU, whose soft segments were built up by PPG, exhibited a glass transition in the range of 40 °C to 60 °C with an inflection point at about 55 °C (Figure 2c). In good agreement with this observation, the loss factor tan δ showed a strong signal at approximately 58 °C in the respective DMA experiment (Figure 2d). Another important aspect was that in between 23 °C and 65 °C, the storage modulus *E*’ of the PEU was greater than that of the foam, thus later allowing controlled transfer of stresses from 4D-printed PEU to the foam. In fact, these requirements were fulfilled as can be seen in Figure 2 (compare Figure 2b with Figure 2d).

**Figure 3:**
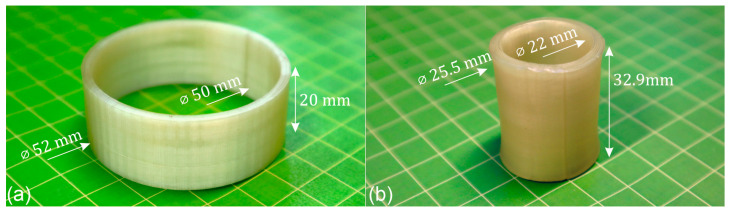
The 4D-printed cylinder made from PEU in its states after FFF (**a**) and after heating to 75 °C and maintaining the temperature for 15 min (**b**).

In preparation for FFF, the synthesized PEU was cryo-milled and extruded into filament [41]. A cylindrical object was then modeled in AutoCAD and subsequently additively manufactured (Figure 3a). Visual inspection of the object produced by FFF revealed that there were no defects. Dimensional characterization with a digital calliper showed a deviation of ± 0.4 mm from the virtual model.

When the object was heated to 75 °C, thus surpassing the *T*_g_ of the PEU (Figure 2c,d), the diameter of the cylinder shrunk while its wall thickness and height increased, as shown in Figure 3b. In detail, a shrinkage of about 51% was observed regarding the outer diameter, which finally measured 25.5 ± 0.6 mm. Beyond that, the cylinder gained in height by 12.9 mm ± 0.3 mm and therefore by approximately 65%. On this basis, a comparison was made with other objects that had already been 4D-printed using the same PEU [41]. Despite different geometries, the abovementioned dimensional changes of the cylinder were close to those of one of our solid cuboids. Indeed, after 4D printing using a nozzle temperature of 165 °C, but a printing speed of 50 mm^−1^, the solid cuboid had a length of 40 mm and a thickness of 2 mm. Upon heating to 75 °C, its length decreased by 51%, accompanied by slight bending, while the thickness increased by 61% [41]. Although the verified change in shape was nominally close to the one of the cylinder, more detailed investigations will be essential to gain a better understanding on similarities regarding shape changes of different 4D-printed objects. In order to reduce the experimental effort, software tools that enable the prediction of the thermoresponsiveness of 4D-printed objects would be desirable, taking into account material-specific parameters.

**Figure 4:**
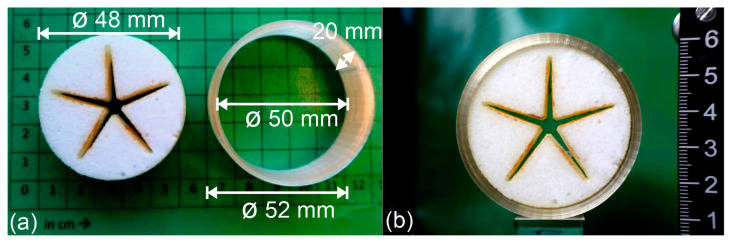
Laser cut SMP foam ((**a**), left) and thermoresponsive tool before assembly ((**a**), right) and after assembly at 23 °C (**b**).

To provide proof of technological feasibility for the transmission of internal stresses from a 4D-printed tool onto an SMP foam, a demonstrator was manufactured (Figure 4). Therefore, a sample of laser cut PEUU foam (Figure 1) and a 4D-printed cylindrical element of PEU were prepared (Figure 4a) and assembled (Figure 4b).

**Figure 5:**
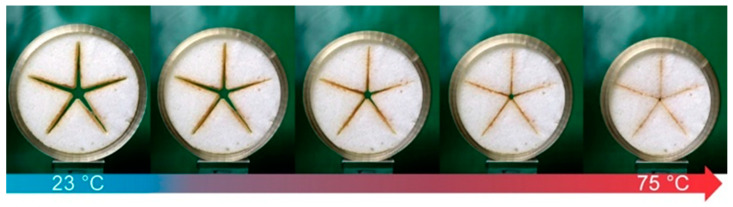
Compression of laser cut PEUU foam by means of a 4D-printed cylinder made of PEU. From left to right, the temperature systematically increased from 23 °C to 75 °C. The right image also corresponds to the state after programming at 23 °C.

Next, the system was steadily heated from 23 °C to 75 °C and the temperature maintained for 15 min. As a result, the foam softened, and the cylinder released its internal stresses by changing its shape, culminating in the reduction of its diameter, whereupon the foam was compressed (Figure 5). Remarkably, the uniform shrinkage caused the star-shaped cut-out of the foam to close. Subsequently, the temperature was lowered to −15 °C to initiate the crystallization of the soft segments of the foam and to cause at least their partial vitrification. The sample was then kept at that temperature for 15 min to stabilize the temporary shape. Upon heating to 23 °C, the foam could be separated from the cylinder. At that time, the outer diameter of the cylinder was 46 mm and its height was 24 mm. The lower shrinkage of the PEU compared with the one documented in Figure 3 can be explained by the establishment of a state of equilibrium, in which the foam resisted further compression.

In view of the pronounced shrinkage of the 4D-printed tool and the associated deformation of the foam, such approaches can also be transferred to other objects with shape memory properties. An essential prerequisite for this is that at a specified deformation temperature, the material used to manufacture the shrink tool has a higher storage modulus compared with the SMP to be deformed. One further practical example are objects that need to be locally compressed, for example a hose with shape memory properties that should be temporarily closed. After filling the hose with a liquid and heating the programmed section of the hose, the original shape will be restored, thus allowing the transport of the liquid. Another scenario is the programming of objects made from SMP containing air vents to realize the concept of programmable heat transfer. In any case, the heating rate can also be used to gain control over the release of internal stresses from a 4D-printed tool, so that predefined deformation rates may be achievable. These depend on the thermal conductivity of the 4D printing material selected, and can be enhanced when using adequate fillers.

**Figure 6:**
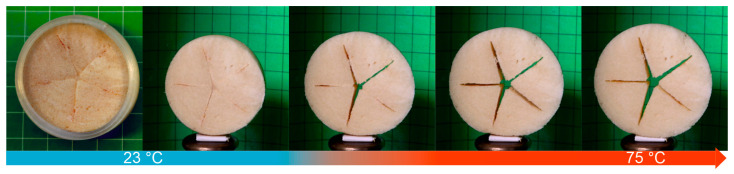
Result of programming a PEUU foam with a 4D-printed cylinder (left image) and thermoresponsiveness of the foam upon gradual heating from 23 °C to 75 °C after storage for 900 days below 30 °C and removal of the cylinder (images from the second picture from left to right).

Being aware of the one-way shape memory effect of the foam [43], the long-term durability of both the cylinder and the programmed foam fixed inside of it was studied. Therefore, the system was stored for 900 days at temperatures below 30 °C. Adjacently, the states after storage and the thermoresponsiveness of the foam were investigated (Figure 6).

The left image in Figure 6 exhibits the sample after the storage period. Remarkably, the 4D-printed cylinder had almost unchanged dimensions, implying a high degree of stability throughout its storage. In fact, its outer diameter was still 46 mm, and its height was 24 mm. After separation from the PEU, the outer diameter of the programmed foam was determined to be about 40 mm. Heating the foam to 75 °C triggered the one-way shape memory effect, as the soft segments of the PEUU melted (Figure 2a). The diameter of the recovered shape of the foam was quantified to be about 47 mm. This shows that the foam was capable of nearly completely recovering its original shape (Figure 4a).

**Figure 7:**
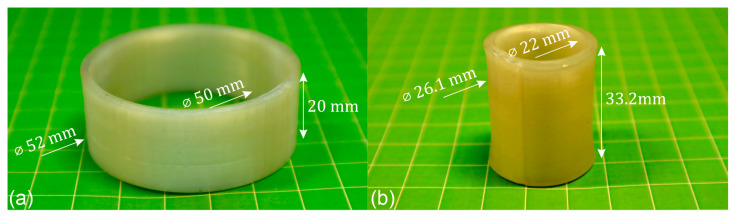
States of a thermoresponsive cylinder made from PEU as obtained from FFF after storing it for 900 days at temperatures below 30 °C (**a**) and after heating to 75 °C, keeping the temperature constant for 15 min and cooling to 23 °C (**b**).

In another experiment, a 4D-printed cylinder made from PEU was stored for 900 days below 30 °C. Even in this thermoresponsive state, it retained its structural integrity (Figure 7a), since the dimensions of the original sample (Figure 3a) did not change. Heating of the cylinder from 23 °C to 75 °C and maintaining this temperature for 15 min resulted in significant shrinkage as exemplified by a decrease in the outer diameter, which finally measured 26.1 mm. In parallel, the height of the cylinder increased drastically until it reached 33.2 mm. Thus, the shape change behavior of the sample was comparable with the one of the freshly 4D-printed sample (compare Figure 3b with Figure 7b). This indicates that 4D-printed objects made from PEU can be stored at temperatures below the *T*_g_ of their soft segments for an extended period of time without losing their thermoresponsiveness. Most remarkably, as shown by another DMA measurement on a PEU sample stored at room temperature for 900 days, only a small increase in *T*_g_ of 4 °C (compare Figure 2d and Figure S1 in ESI) occurred, which could be due to physical aging [46,47,48], since the PEU was stored with its switching segments being in a glassy state, while slight changes in temperature were allowed to occur, favoring the formation of non-equilibrium states. Nonetheless, the material functionality remained intact.

## 4. Conclusions

This work demonstrates the practical feasibility of manufacturing a cylinder via 4D printing, and using it to compress the lateral surface of a cylindrical sample of a laser cut foam with shape memory properties as triggered by heating. Subsequent cooling led to the fixation of the foam in its new shape. Therefore, the 4D-printed object could be used as a programming tool. The concept of controlled release of internal stresses as introduced via 4D printing may not be limited to the programming of SMP foam, but can also offer a possibility to program other SMPs with geometrically challenging dimensions. In general, such approaches, may even be extended by utilization of 4D printing materials with other specific thermomechanical properties, modification of the shape of 4D-printed tools and adaptation of the manufacturing process by varying the printing parameters to alter the distribution of internal stresses.

This suggests that such thermoresponsive tools offer a wide variety of designs both in terms of geometry and functionality. Interestingly, stress relaxation processes seemed to be negligible under room temperature conditions, since internal stresses could be retained in the 4D-printed object for at least 900 days. In this respect, after prolonged storage, the “shrinking on command” of a 4D-printed object can be understood as bringing it out of hibernation. In addition, the foam was also able to return almost completely to its original shape.

In the future, the digitization of shape changes seems indispensable for accurate predictions about the material behavior of 4D-printed objects. Ideally, machine learning can be integrated into the software algorithms leading to an autonomous 4D printing technology [19,49], that permits drawing conclusions on potential shape changes of 4D-printed objects in advance of AM. However, it should not be forgotten that the thermal properties and the deformation behavior of its counterpart, namely the SMP, to which the force will be transferred, must also be taken into account.

The technological significance of 4D-printed tools may increase further when they are applied to other programmable materials like hydrogels, etc. [50,51,52,53,54,55,56,57,58,59]. Above all, however, they may also assist in construction technology and be part of design for disassembly concepts. Additionally, due to the ease with which 4D-printed objects can be produced and the uncomplicated temperature control, they could even facilitate approaches to scaling the programming of SMPs when many programming tools are printed at once. Nevertheless, the sustainability of the PEU used represents a further advantage, as the thermoresponsiveness decreases only slightly if it is mechanically recycled after 4D printing [41].

## Data Availability

Data are contained within the article and Supplementary Materials.

## Supplementary Materials

The following supporting information can be downloaded at: https://www.mdpi.com/article/10.3390/polym16101393/s1, Figure S1: Thermomechanical properties of PEU as determined by DMA (temperature dependence of storage modulus E’, solid line and loss factor tan δ, dashed dotted line). A heating rate of 3 °C × min^−1^ was selected.
