# Supplementary material for: 4D-Printed Tool for Compressing a Shape Memory Polyurethane Foam during Programming
Source: Polymers (Basel). 2024 May 14;16(10):1393. doi: 10.3390/polym16101393 (PMC11124973; doi:10.3390/polym16101393)
Supplement: Supplementary file 1 [file polymers-16-01393-s001.zip › polymers-2940658-supplementary.pdf]

Electronic Supplementary Information

4D-Printed Tool for Compressing A Shape Memory Polyurethane Foam during Programming

Dilip Chalissery and Thorsten Pretsch *

Fraunhofer Institute for Applied Polymer Research IAP, Geiselbergstraße 69, 14476 Potsdam, Germany; dilip.chalissery@iap.fraunhofer.de
* Correspondence: thorsten.pretsch@iap.fraunhofer.de

The thermomechanical properties of a PEU sample, which was stored for more than 900 days under room temperature conditions, were characterized using DMA. A slight increase in T_g (compare Figure 2d and ESI S1), could be observed.

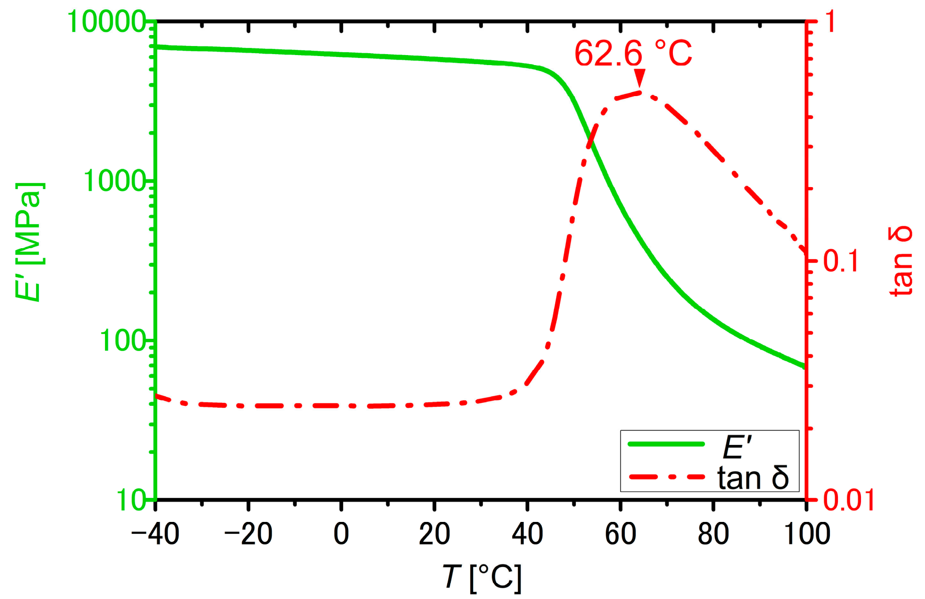

Figure S1. Thermomechanical properties of PEU as determined by DMA (temperature dependence of storage modulus E′, solid line, and loss factor tan δ, dashed dotted line). A heating rate of 3 °C × min^{-1} was selected.
